# Construction of a Candidate Differentially Expressed Transcript Profile Associated with the Hair Follicle Cycle in Jiangnan Cashmere Goats (*Capra hircus*)

**DOI:** 10.3390/biology15060498

**Published:** 2026-03-20

**Authors:** Cuiling Wu, Gvlnigar Amar, Sen Tang, Asma Anwar, Yaqian Wang, Wenna Liu, Qingfa Yan, Shengchao Ma, Xuefeng Fu

**Affiliations:** 1 Xinjiang Key Laboratory of Special Species Conservation and Regulatory Biology, International Center for the Collaborative Management of Cross-Border Pest in Central Asia, College of Life Science, Xinjiang Normal University, Urumqi 830054, China; cuiling_wu@163.com (C.W.); 13579812147@163.com (G.A.); v2762636615@163.com (Q.Y.); 2 Xinjiang Key Laboratory of Animal Biotechnology, Xinjiang Key Laboratory of Reproductive Regulation and Breeding for Ruminant Livestock, Key Laboratory of Genetic Breeding and Reproduction of Herbivorous Livestock of Ministry of Agriculture and Rural Affairs, Xinjiang Uygur Autonomous Region Academy of Animal Science, Urumqi 830011, China; tangsensen610@163.com (S.T.); asma247462@163.com (A.A.); wangyaqlan@163.com (Y.W.); lwn2362@163.com (W.L.)

**Keywords:** cashmere goats, hair follicle cycle, DETs, intradermal fat deposition, AS patterns

## Abstract

Dynamic changes in gene and transcript expression represent a key factor in regulating the cyclical development of hair follicles. In this study, based on Nanopore sequencing (ONT-seq) data of skin tissue from three developmental stages (anagen (An), catagen (Cn), and telogen (Tn)) of Jiangnan cashmere goat hair follicles, this study presents a profile of candidate DETs implicated in cycle regulation by delineating their stage-specific expression patterns and dynamic expression trends from anagen to telogen. A large proportion of the candidate DETs were significantly enriched in functions related to fat synthesis, storage, or metabolism, with significance levels of *p* < 0.05 or *p* < 0.01. These significantly enriched DETs, which were generally upregulated from An to Cn or downregulated from Cn to Tn, support a model where accelerated intradermal fat deposition promotes the progression from An to Cn, while its subsequent decrease facilitates the transition from Cn to Tn. Concurrently, our results also suggest a potential role for dynamic changes in AS patterns in regulating the hair follicle cycle. This regulatory role of AS patterns is potentially mediated through affecting genes related to lipid synthesis/metabolism or cell structure/interaction. Notably, a broader range of fat synthesis, storage, or metabolism-related transcripts showed significant differential expression (*p* < 0.05) in the An vs. Cn group. Ultimately, by establishing this candidate DET profile, we aim to provide fresh perspectives for deciphering the complex molecular regulation of the hair follicle cycle and to identify new targets for genetically enhancing or molecularly breeding cashmere traits in cashmere goats.

## Simple Summary

This study utilized Nanopore sequencing technology to analyze the transcriptomic data of skin tissues from Jiangnan cashmere goats during the anagen, catagen, and telogen stages of hair follicles. Through the analysis of differentially expressed transcripts (DETs) and alternative splicing (AS) events, we revealed the association between the expression dynamics of candidate DETs and the hair follicle cycle. The results demonstrated that a large number of transcripts related to fat synthesis, storage, or metabolism exhibit stage-specific expression patterns, and their dynamic changes are closely linked to the periodic fluctuations in intradermal fat deposition. Simultaneously, dynamic alterations in alternative splicing patterns may play a potential role in regulating the hair follicle cycle. The candidate DET profile established in this study provides new insights into deciphering the complex molecular regulatory mechanisms of the hair follicle cycle and offers potential targets for the genetic improvement and molecular breeding of cashmere goat traits.

## 1. Introduction

The cashmere goat is an important economic livestock species in Xinjiang, China. Its cashmere, known for its soft texture, high luster, and excellent warmth retention, holds significant economic value and serves as a crucial raw material for high-end textile industries [1]. It is acclaimed as “soft gold” [1]. However, the cashmere goat industry in Xinjiang still faces numerous challenges. For instance, the yield of cashmere remains relatively low, key quality traits such as fiber fineness, length, and uniformity are still unstable, and the genetic improvement of cashmere-related traits has been progressing slowly [1]. These factors constrain the sustainable development of the cashmere industry in the Xinjiang region [1]. Applying molecular breeding technology to enhance cashmere yield and quality stability will significantly boost the profitability and competitiveness of the cashmere goat industry, meeting the growing domestic and international demand for high-quality cashmere. In cashmere goats, hair follicles serve as the fundamental units for cashmere production [1]. Their cyclic activity directly dictates the follicular structure, consequently determining critical economic traits including fiber growth rate, diameter, and length [2]. Unraveling the molecular mechanisms governing the hair follicle cycle in cashmere goats will identify key targets for molecular breeding, thus facilitating the cultivation of elite genetic strains characterized by enhanced cashmere production, reduced fiber diameter, and improved uniformity.

From a molecular biological perspective, the execution of gene function is a central tenet in the molecular regulation of the hair follicle cycle in animals. It governs the precise control of critical biological processes, including the activation of hair follicle stem cells, the proliferation and differentiation of matrix cells, and the keratinization of the fiber, which collectively dictate the efficiency of transitions within the hair follicle cycle. A substantial body of research has uncovered a number of candidate genes implicated in the hair follicle cycle. Key examples include *WNT* and *HOXC* genes, which regulate follicle regeneration or initiation [3,4]; *β-catenin*, *SHH*, *BMP*, and *Notch* genes, which control morphogenesis or stem cell differentiation [5,6,7,8]; *FGF* and *IGF* genes, which govern hair growth [9,10]; *TGF-β*, which modulates the hair follicle cycle itself [11]; and *KRT* and *Noggin*, which are responsible for basic hair structure and the regulation of follicle development [12,13,14]. Nevertheless, the genes currently known are merely constituent parts of the intricate regulatory network underlying hair follicle development and periodic cycling. The discovery of a broader repertoire of functional candidate genes, along with the elucidation of their interactive molecular mechanisms, necessitates further systematic exploration and validation.

It is particularly important to note that the functional realization of genes relies on the crucial step of transcription into transcripts, in which AS plays a vital regulatory role [15]. As a highly conserved post-transcriptional regulatory mechanism, alternative splicing enables a single gene to generate multiple transcript isoforms through different splicing patterns, thereby significantly increasing the diversity and functional complexity of the proteome [15]. Recent studies have shown that AS of certain genes (such as *APC*, *POFUT1*, *TGFBR3*, and *FGF5* genes) dynamically regulates biological processes including hair follicle stem cell activation and hair follicle cycle transition by controlling the ratio and expression patterns of different transcripts [16,17]. These findings indicate that alternative splicing could play a key role in the hair follicle cycle regulatory network. Consequently, elucidating the role of alternative splicing in regulating the hair follicle cycle will offer fresh insights into the molecular mechanisms underlying hair regeneration. In contrast to the extensive screening of hair follicle cycle-related candidate genes, systematic studies on alternative splicing events in hair follicle development and cycle regulation remain relatively limited.

Traditionally, the study of AS has been dominated by second-generation transcriptome sequencing [18,19,20,21,22]. This approach leverages short-read data in conjunction with bioinformatic algorithms to infer splicing events [18,19,20,21,22]. However, this approach suffers from inherent limitations when it comes to precisely resolving complex splice isoforms, discriminating between highly similar splicing variants, and determining full-length transcript architectures [23]. In contrast to second-generation sequencing, third-generation sequencing (e.g., ONT-seq), with its long-read capability, directly captures full-length transcripts, enabling accurate identification of alternative splicing combinations without assembly and excelling in detecting complex isoforms, fusion genes, and low-frequency splicing events [24]. These advantages make third-generation sequencing a superior choice for investigating transcriptome complexity and the dynamic regulation of AS.

This study focused on the Jiangnan cashmere goat, an important breed in the Xinjiang region. Skin tissue samples were collected from the hair follicles of these goats at distinct developmental stages (An, Cn, and Tn) for ONT-seq. The obtained data were subjected to DET analysis, differential AS event analysis, and functional enrichment analysis. This integrated approach facilitated the identification of key DETs and AS events among the three stages, leading to the establishment of a gene/transcript profile associated with hair follicle cycle regulation in cashmere goats. Furthermore, during the initial phase of this study, we also conducted proteomic and metabolomic analyses on these samples. Using statistical and metabolomic analytical approaches, we identified metabolites and proteins associated with the hair follicle cycle (for references, please refer to Section 2.3.4). We further integrated the ONT-seq data obtained in this study with the previously acquired proteomic and metabolomic data to construct a multi-omics interaction network. The findings of this study provide novel perspectives on the complex molecular regulation mechanisms governing the hair follicle cycle, thereby contributing to the identification of new targets for the genetic improvement of cashmere traits in cashmere goats.

## 2. Materials and Methods

### 2.1. Collection of Skin Tissue Samples

Six 24-month-old female Jiangnan cashmere goats with comparable physiological status were selected as experimental subjects for this study, with three individuals sourced from each of the Kechuang Breeding Center (Qitai, Xinjiang, China) and the Baihutai Cashmere Goat Breeding Center (Aksu, Xinjiang, China). Skin samples were collected from these six goats at three distinct stages of the hair follicle cycle: An (September, sample size (n) = 6), Cn (January, n = 6), and Tn (March, n = 6) [1]. All three skin tissue samples from the different time periods are longitudinal samples (same individuals across An, Cn, and Tn). Before sample collection, six cashmere goats were subjected to local anesthesia and hair removal from the skin tissue at the shoulder blades. Subsequently, a skin sample of 1 cm^2^ was collected using a skin sampler with a diameter of 10 mm. Following sample collection, the skin wound on each goat was disinfected, and hemostasis was achieved. Appropriate post-procedural care was then provided. Finally, the skin tissue samples were rinsed with phosphate-buffered saline (PBS; Thermo Fisher Scientific Inc., Waltham, MA, USA), aliquoted into cryovials, and stored at −80 °C, resulting in a total of 18 samples. This study was carried out in compliance with all relevant ethical regulations, and all animal experiments were approved by the Animal Ethics Committee of the Xinjiang Uygur Autonomous Region Academy of Animal Science.

### 2.2. Extraction of Total RNA, Construction of Sequencing Libraries and Sequencing

Total RNA was extracted from all 18 skin tissue samples using Trizol reagent (Invitrogen, Carlsbad, CA, USA), strictly following the manufacturer’s instructions. Following extraction, the OD260/280 and OD260/230 ratios of the total RNA were measured using a NanoDrop 2000 (Thermo Fisher Scientific Inc., Waltham, MA, USA). The RNA integrity was assessed by the RNA Integrity Number (RIN) obtained with an Agilent 2100 Bioanalyzer (Agilent Technologies Inc., Santa Clara, CA, USA). The quality assessment results demonstrated that all 18 total RNA samples had OD260/280 ratios between 1.8 and 2.0, OD260/230 ratios greater than 2.0, and RIN values greater than 8.0, confirming their suitability for sequencing library construction.

A quantity of 1 ug of total RNA was used for cDNA library construction following the cDNA-PCR Sequencing Kit (SQK-LSK110 and EXP-PCB096) protocol provided by Oxford Nanopore Technologies (Oxfordshire, UK). The template-switching activity of reverse transcriptase enriched for full-length cDNAs and appended predefined PCR adapters directly to both ends of the first-strand cDNA. The cDNA was then amplified by PCR for 14 cycles using LongAmp Taq polymerase (NEB, Ipswich, MA, USA). The resulting PCR products were subsequently ligated with ONT adapters using T4 DNA ligase (NEB, Ipswich, MA, USA). DNA purification was performed using Agencourt XP beads (Beckman Coulter, Inc., Brea, CA, USA) according to the ONT protocol. The final cDNA libraries were loaded onto FLO-MIN109 flow cells and sequenced on the PromethION platform at Biomarker Technologies Corporation (Beijing, China).

### 2.3. Data Analysis

#### 2.3.1. Preprocessing of ONT-seq Data

Raw reads were first filtered with minimum average read quality score = 6 and minimum read length = 350 bp. Ribosomal RNA was discarded after mapping to rRNA database (Silva database, https://www.arb-silva.de/, accessed on 5 December 2024). Next, full-length non-chemiric (FLNC) transcripts were determined by searching for primers at both ends of reads. Quality control was performed on the sequencing data using NanoPlot (v 1.44.0). The Q-score for each dataset was calculated using NanoPlot, which derives quality scores from the Phred-scaled probabilities generated during base calling. Clusters of FLNC transcripts were obtained after mapping to goat reference genome (ARS1.2, GCA_001704415.2) with mimimap2 (v 2.28) [25], and consensus isoforms were obtained after polishing within each cluster by pinfish (v 0.1.0). Consensus sequences were mapped to goat reference genome using minimap2 (v 2.28). Mapped reads were further collapsed by cDNA_Cupcake package (v 23.0.0) with min-coverage = 85% and min-identity = 90%. 5′ sequence differences were not considered when collapsing redundant transcripts. The expression level of transcripts was measured by Counts Per Million (CPM). The formula for calculating CPM is: CPM = (Number of reads mapped to the transcript/Total effective reads of the sample) × 1,000,000.

#### 2.3.2. Structure Analysis

Transcripts were validated against known reference transcript annotations with gffcompare (v 0.12.6) [26]. AS events including intron retention, exon skipping, alternative 3′ splice site, alternative 5′ splice site, and mutually exclusive exon were identified by the AStalavista tool (v 4.0.1) [27]. Differential AS events across the various hair follicle developmental stages were analyzed using the PSI-Sigma software (v 1.1), which calculated the delta Percent Spliced In (delta PSI) values for these events. The *p*-value for alternative splicing events was derived from a two-sample *t*-test. The False Discovery Rate (FDR) was calculated by applying multiple hypothesis testing correction using the Benjamini–Hochberg method. The screening criteria for significant differential alternative splicing events were set as: delta PSI > 0.1, *p* < 0.01, and FDR < 0.05.

#### 2.3.3. DET Analysis and Functional Annotation Analysis

Differential expression analysis was performed at the transcript level. DET analysis at different developmental stages was performed using the DESeq2 R package (1.6.3) [28,29,30,31]. The resulting *p*-values were adjusted using the Benjamini and Hochberg’s approach for controlling the FDR. Genes with a *p*-value < 0.01 and fold change ≥ 1.5 found by DESeq2 were assigned as differentially expressed. Gene Ontology (GO) and KEGG pathway enrichment analyses were performed using the DAVID database (https://davidbioinformatics.nih.gov/, accessed on 4 March 2025). Terms and pathways with a *p*-value < 0.05 and FDR < 0.05 were considered statistically significant.

#### 2.3.4. Multi-Omics Joint Analysis

In the integrated analysis of transcriptomics, proteomics, and metabolomics, the transcriptomics data were derived from this study, while both the proteomics [32] and metabolomics [33] data were obtained from our previous research. The transcriptomic data in this study were derived from the same batch of individuals as the proteomic and metabolomic data. It should be particularly emphasized that Wu et al.’s study utilized the proteomic data of five out of these six individuals [32]. The data of the six individuals were obtained at the same time. We have supplemented the proteomic data of An, Cn, and Tn of the unused individual into this joint analysis. Based on the differentially expressed genes (DEGs), differentially expressed proteins (DEPs), and differentially expressed metabolites (DEMs) related to the hair follicle cycle identified from the aforementioned data, an interaction network was constructed. The method and threshold for screening DEGs refer to Section 2.3.4, while the methods and thresholds for screening DEPs and DEMs refer to the studies by Wu et al. [32] and Ma et al. [33], respectively. The Spearman method was used to analyze the correlations among the DEGs, DEPs, and DEMs. Significant relationships were filtered using the criteria: |coefficient| > 0.80 and *p* < 0.05. Finally, the interaction network was generated using the Weishengxin website (www.bioinformatics.com.cn, accessed on 10 May 2025).

## 3. Results

### 3.1. Quality Control Results of Sequencing Data

After completing the ONT-Seq of 18 skin samples from Jiangnan cashmere goats, the 18 raw datasets obtained were filtered to produce 18 clean datasets. The clean reads of these 18 datasets ranged from 4,595,239 to 7,358,680, with total bases ranging from 8,694,667,258 to 5,929,107,441. The N50 values fell between 935 and 1653, mean read lengths ranged from 887 to 1363, and maximum read lengths spanned from 31,257 to 219,565. N50 is defined as follows: all sequenced reads are sorted by length from longest to shortest, and their lengths are cumulatively summed. When the cumulative sum reaches 50% of the total length of all sequencing data, the length of the last read added at that point is the N50 value.

The mean Q-score for all datasets was Q12. These results collectively demonstrate the high quality of the 18 sequenced datasets, confirming their suitability for subsequent analysis. It should be noted that the genome.bed file in the Supplementary Materials contains detailed information for sequences identified by their ONT accession numbers.

### 3.2. Screening of Hair Follicle Cycle-Related DETs

#### 3.2.1. DET Analysis Results

**Figure 1:**
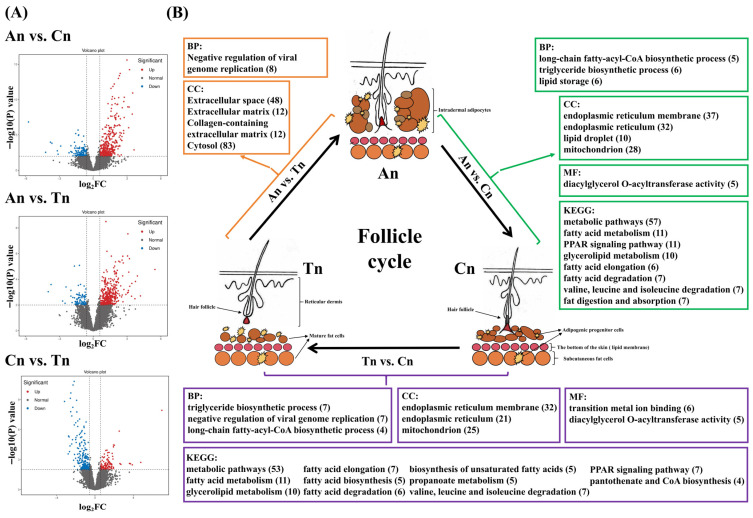
The results of DET analysis and functional enrichment analysis of DET among different developmental stages of hair follicle. (**A**) The volcano plot of the DET analysis results. The X-axis shows the Log_2_FC value of transcripts, and the Y-axis shows the −log_10_(P) value of transcripts. (**B**) The GO and KEGG enrichment analysis results of DET. The figure only shows all significant GO entries and KEGG pathways in different comparison groups. The genes corresponding to DETs were used for GO and KEGG enrichment analysis. The number of genes enriched in each entry or pathway is shown in parentheses. The figure also reflects the deposition status of fat in the skin at different stages of hair follicle development.

DET analysis revealed 510 (132 down-regulated, 378 up-regulated, annotated to 321 genes), 510 (94 down-regulated, 416 up-regulated, annotated to 403 genes), and 324 (218 down-regulated, 106 up-regulated, annotated to 156 genes) DETs in the An vs. Cn, An vs. Tn, and Cn vs. Tn groups, respectively (Figure 1A, Tables S1–S3).

#### 3.2.2. GO and KEGG Enrichment Analysis of DET

GO and KEGG enrichment analyses of the genes annotated in Section 3.2 revealed that in the An vs. Cn group, 37, 57, 32, 11, 11, 10, 10, 6, 7, 5, 7, 7, 5, 6, 6, and 28 genes were respectively enriched into endoplasmic reticulum membrane (GO:0005789), Metabolic pathways (chx01100), endoplasmic reticulum (GO:0005783), Fatty acid metabolism (chx01212), PPAR signaling pathway (chx03320), lipid droplet (GO:0005811), Glycerolipid metabolism (chx00561), Fatty acid elongation (chx00062), Fatty acid degradation (chx00071), long-chain fatty acyl-CoA biosynthetic process (GO:0035338), Valine leucine and isoleucine degradation (chx00280), Fat digestion and absorption (chx04975), diacylglycerol O-acyltransferase activity (GO:0004144), triglyceride biosynthetic process (GO:0019432), lipid storage (GO:0019915) and mitochondrion (GO:0005739) (*p* < 0.05 and FDR < 0.05) (Figure 1B and Table S4).

In the An vs. Tn group, 8, 48, 12, 12, and 83 genes were respectively enriched into negative regulation of viral genome replication (GO:0045071) and extracellular space (GO:0005615), extracellular matrix (GO:0031012), collagen-containing extracellular matrix (GO:0062023) and cytosol (GO:0005829) (*p* < 0.05 and FDR < 0.05) (Figure 1B and Table S5).

In the An vs. Cn group, 32, 53, 11, 10, 7, 7, 6, 7, 5, 21, 25, 5, 7, 6, 5, 5, 7, 7, 4, and 4 genes were respectively enriched into endoplasmic reticulum, membrane (GO:0005789), Metabolic pathways (chx01100), Fatty acid metabolism (chx01212), Glycerolipid metabolism (chx00561), Fatty acid elongation (chx00062), triglyceride biosynthetic process (GO:0019432), transition metal ion binding (GO:0046914), negative regulation of viral genome replication (GO:0045071), diacylglycerol O-acyltransferase activity (GO:0004144), endoplasmic reticulum (GO:0005783), mitochondrion (GO:0005739), Fatty acid biosynthesis (chx00061), Valine leucine and isoleucine degradation (chx00280), Fatty acid degradation (chx00071), Biosynthesis of unsaturated fatty acids (chx01040), Propanoate metabolism (chx00640), PPAR signaling pathway (chx03320), long-chain fatty-acyl-CoA biosynthetic process (GO:0035338), and Pantothenate and CoA biosynthesis (chx00770) (*p* < 0.05 and FDR < 0.05) (Figure 1B and Table S6).

**Figure 2:**
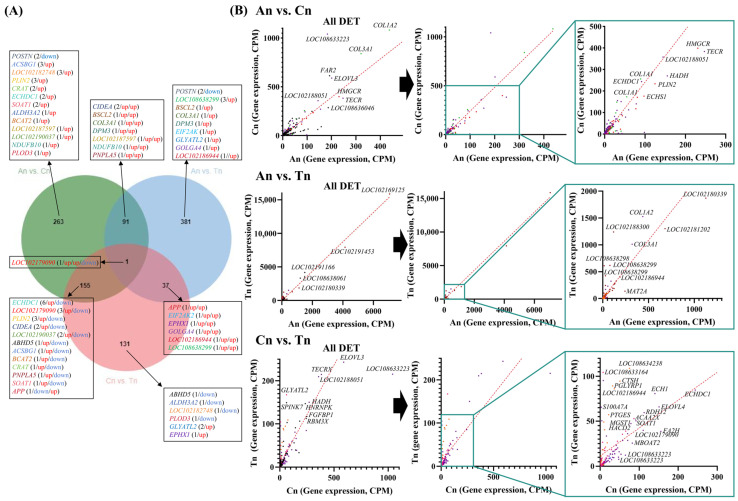
Analysis results of the expression characteristics of DET related to the hair follicle cycle. (**A**) Venn diagram showing the number of differentially expressed genes in the specified groups. The Venn diagram highlights the genes enriched in significant terms or pathways. Within the parentheses, the number of types of DETs representing each gene, along with their expression patterns in An vs. Cn, An vs. Tn, Cn vs. Tn, An vs. Cn/An vs. Tn (the intersection of the An vs. Cn and the An vs. Tn groups), An vs. Cn/Cn vs. Tn (the intersection of the An vs. Cn and the Cn vs. Tn groups), An vs. Tn/Cn vs. Tn (the intersection of the An vs. Tn and the Cn vs. Tn groups), or An vs. Cn/An vs. Tn/Cn vs. Tn (the intersection of the three comparison groups). Here, “up” indicates up-regulated expression, and “down” indicates down-regulated expression. (**B**) Expression patterns of DETs during different hair follicle developmental stages. The scatter plot displays some DETs represented by specific data points. DETs represented by colored points are involved in significantly enriched pathways or terms. Red points represent DETs exclusively found in the An vs. Cn group, dark red points represent DETs exclusively found in the An vs. Tn group, pink points represent genes exclusively found in the Cn vs. Tn group, green points represent DETs found in the intersection of the An vs. Cn and An vs. Tn groups, purple points represent DETs found in the intersection of the An vs. Cn and Cn vs. Tn groups, orange points represent DETs found in the intersection of the An vs. Tn and Cn vs. Tn groups, and blue points represent DETs found in the intersection of all three comparison groups.

Venn diagram analysis revealed that the intersection between the An vs. Cn group and the An vs. Tn group contained 92 common DETs, the intersection between the An vs. Cn group and the Cn vs. Tn group contained 156 common DETs, and the intersection between the An vs. Tn group and the Cn vs. Tn group contained 38 common DETs. One common DET was found in the intersection of all three groups (Figure 2A). In the comparison between the An and Cn groups, the expression level of DETs (these DETs were significantly enriched in the GO terms and KEGG pathways shown in Figure 1B) was higher in Cn, and similarly, in the comparison between the Cn and Tn groups, it was also higher in Cn (Figure 2B).

### 3.3. Bioinformatics Analysis of AS Events

#### 3.3.1. Statistics of AS Events at Different Stages of Hair Follicle Development

**Figure 3:**
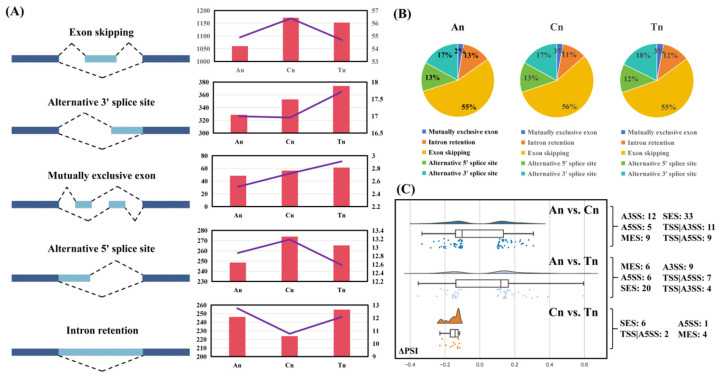
Analysis results of AS events at different stages of hair follicle development. (**A**) Types of as events detected in skin tissue sequencing data. In the combined bar and line chart, the left bar chart’s Y-axis shows the number of occurrences for each type of AS event, while the right line chart’s Y-axis shows the proportion of each type of AS event relative to the total number of AS events. (**B**) The pie chart displays the proportion of each of the five types of AS events relative to the total number of AS events. (**C**) Results of differential AS event analysis. The number and type distribution of significant differential alternative splicing events identified in the different hair follicle developmental stage comparison groups (An vs. Cn, An vs. Tn, and Cn vs. Tn) are presented. It includes the counts of various splicing event types in each comparison group and illustrates the distribution of ΔPSI values for the AS events through box plots. Box plot shows the mean ± SEM of ΔPSI values in three comparison groups.

AS events were identified in samples from each developmental stage. The detected splicing types included: exon skipping, alternative 3′ splice site, alternative exon, alternative 5′ splice site, and intron retention (Figure 3A). Exon skipping exhibited the highest frequency across all three stages, followed by alternative 5′ splice site, alternative 3′ splice site, and intron retention (Figure 3B). The relative proportions of different types of AS events fluctuate among An, Cn, and Tn, but the overall variation is relatively small. Specifically, the frequency of exon skipping initially increased then decreased, while alternative 5′ splice site usage showed a declining trend. Conversely, alternative 3′ splice site and alternative exon demonstrated increasing trends, and intron retention frequency initially decreased before rising (Figure 3A,B).

#### 3.3.2. Analysis of Differential AS Events

The analysis of differential AS events revealed that in the An vs. Cn, An vs. Tn, and Cn vs. Tn groups, 79 (including 12 alternative 3′ splice site (A3SS), 5 alternative 5′ splice site (A5SS), 9 Multi-exon skipping (MES), 33 Single-exon skipping (SES), 11 TSS|A3SS, and 9 TSS|A5SS, involving 64 genes), 52 (20 SES, 9 A3SS, 7 TSS|A5SS, 6 MES, 6 A5SS, and 4 TSS|A3SS, involving 39 genes), and 13 (1 A5SS, 4 MES, 6 SES, and 2 TSS|A5SS, involving 10 genes) significant AS events were identified, respectively (Figure 3C, Tables S7–S9).

**Figure 4:**
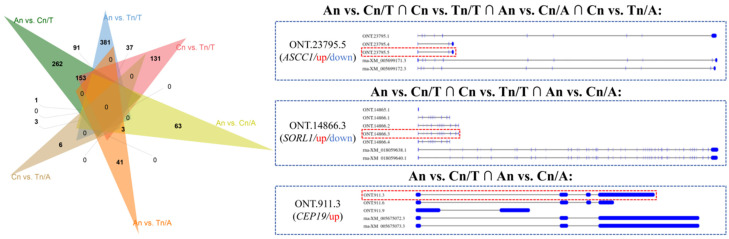
Intersection analysis of DETs and differential AS events related to the hair follicle cycle. Note: The Venn diagram shows the overlap of transcripts identified as DETs and/or involved in differential AS events among three comparison groups (An vs. Cn, An vs. Tn, Cn vs. Tn). Three key transcripts—ONT.23795.5 (*ASCC1*), ONT.14866.3 (*SORL1*), and ONT.911.3 (*CEP19*)—are highlighted because they appear in both DET and differential AS analyses. The expression pattern of this transcript in the An vs. Cn/Cn vs. Tn (*ASCC1* and *SORL1* genes) or the An vs. Cn (*CEP19* gene) is shown in this figure. “up” indicates upregulated expression, and “down” indicates downregulated expression.

Venn diagram analysis revealed that the intersection among the An vs. Cn/T (/T:/Transcript), Cn vs. Tn/T, An vs. Cn/A (/A:/Alternative Splicing), and Cn vs. Tn/A groups contained one common transcript: ONT.23795.5 (*ASCC1* gene). The intersection among the An vs. Cn/T, Cn vs. Tn/T, and An vs. Cn/A groups contained one common transcript: ONT.14866.3 (*SORL1* gene). Finally, the intersection between the An vs. Cn/T and An vs. Cn/A groups also contained one common transcript: ONT.911.3 (*CEP19* gene) (Figure 4). We visualized the structure of each transcript of the *ASCC1*, *SORL1*, and *CEP19* genes using sashimi plots (Figure 4).

### 3.4. Construction of Multi-Omics Interaction Networks Based on Transcriptomic, Proteomic, and Metabolomic Data

**Figure 5:**
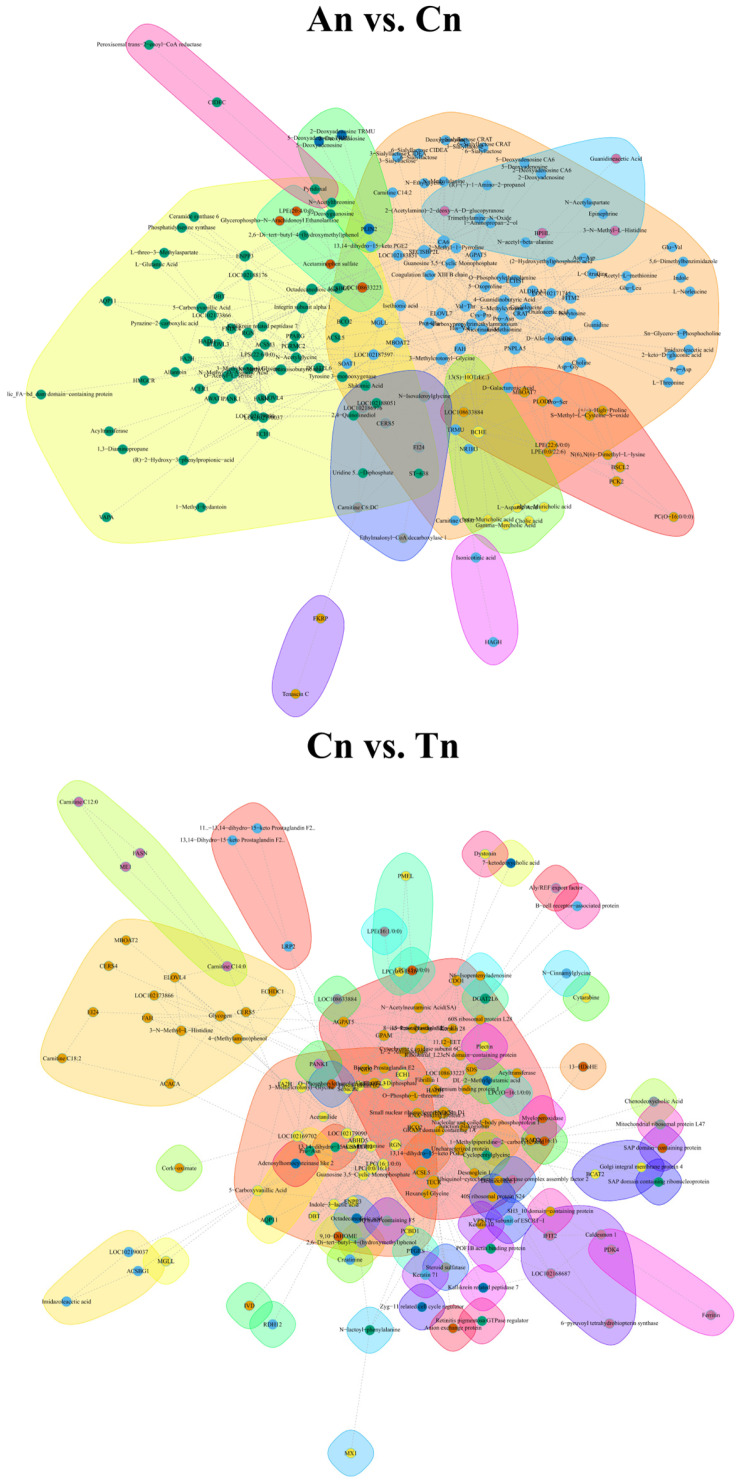
Shows the interaction network of DEG, DEP and DEM related to hair follicle cycle. Note: Only the top 500 interaction pairs ranked by |coefficient| values are displayed in the figure. To distinguish DEGs from DEPs, DEPs are represented by numbers.

Through multi-omics integrative analysis, we constructed a correlation network diagram related to DEGs, DEPs, and DEMs during the hair follicle cycle in Jiangnan cashmere goat (Figure 5). In the An vs. Cn group, 5095 significant relationship pairs were identified (Table S10), while in the Cn vs. Tn group, 1952 significant relationship pairs were obtained (Table S11). These significant correlations suggest potential interactions among the genes, proteins, and metabolites located within these relationship pairs. It is worth noting that the interaction network includes genes (Figure 2) that were significantly enriched in GO terms and KEGG pathways (Figure 1).

## 4. Discussion

In this study, based on ONT-seq, we identified 510, 510, and 324 DETs between An vs. Cn, An vs. Tn, and Cn vs. Tn, respectively, were potentially related to the hair follicle cycle changes in Jiangnan cashmere goats. Multi-omics joint analysis revealed that, compared to the Cn vs. Tn group, the interactions/connections between genes, as well as between genes, proteins, and metabolites, were more active in the An vs. Cn group. This finding aligns with the phenomenon of rapid hair follicle development during the An [1]. Previously, based on the Illumina sequencing platform, Wu et al. identified 21 key genes related to the hair follicle cycle in Jiangnan cashmere goats, including *S100A7A*, *FA2H*, *LOC102190037*, *LOC102179090*, *LOC102173866*, *KRT2*, among other genes [34]. Similarly, Wang et al., also based on second-generation RNA-seq data, identified 12 candidate genes associated with the hair follicle cycle in Inner Mongolia cashmere goats, including *COL1A1*, *COL1A2*, *AQP3*, *FA2H*, and other genes [35]. Our research results also support the conclusions of Wu et al. and Wang et al., as the aforementioned candidate genes were likewise identified in our study [34,35]. We present the expression profiles of these genes at the transcript level and ultimately established a candidate DET profile related to hair follicle (Jiangnan cashmere goats hair follicle). Within this candidate DET profile, we annotated the fold change values of some transcripts from the second-generation sequencing data analysis by Wu et al. to corroborate the relative accuracy of the findings in this study (Tables S1–S3) [34]. The differences between the results of this study and those of Wu et al. may be related to differences in sample size, batch effects, and the superiority of ONT-seq over second-generation transcriptome sequencing in transcript identification. The additional candidate DETs potentially associated with the hair follicle cycle discovered in this research can provide new insights for further in-depth exploration of the molecular regulatory mechanisms underlying the hair follicle cycle in cashmere goats.

Previous studies have shown that dermal white adipose tissue (WAT) undergoes synchronous changes with the hair follicle cycle. Specifically, during An, as the hair follicle elongates downward, the dermal WAT layer thickens significantly, with lipid-filled mature dermal adipocytes surrounding the follicle [36,37]. The volumetric expansion of WAT results from both the proliferation and differentiation of dermal preadipocytes and the hypertrophy of mature dermal adipocytes [36,37]. In Cn, the follicle undergoes apoptosis and upward contraction, reducing the dermal WAT layer thickness to half of its maximum [38,39,40,41,42]. During Tn, the follicle remains small and inactive, with the layer of mature dermal adipocytes diminishing to a single layer (Figure 1B) [38,39,40,41,42]. In this study, in the An vs. Cn group, DETs were significantly enriched in pathways and functional terms related to lipid synthesis, metabolism, or storage. During the transition from An to Cn, the majority of these DETs were upregulated, indicating enhanced lipid synthesis. In contrast, in the Cn vs. Tn group, the number of DETs associated with lipid synthesis, metabolism, or storage decreased, and most were downregulated during the transition from Cn to Tn, suggesting a weakening of lipid synthesis. The expression patterns of lipid synthesis-related DETs revealed in this study align with the previously reported cyclical content changes of WAT during the hair follicle cycle. In summary, we used ONT-seq to confirm these morphological observations at the molecular level. We further propose that the cumulative increase in the expression of lipid-related genes during the transition from An to Cn promotes hair follicle growth and the shift from An to Cn, whereas the decline in the expression of these genes facilitates the transition from Cn to Tn. DETs associated with fat synthesis, storage, or metabolism may play a critical role in regulating the hair follicle.

The initial RNA transcript of a gene can yield multiple distinct mature mRNA transcripts through different splicing mechanisms, ultimately translating into a variety of functionally related yet different proteins, thereby fulfilling the gene’s functions. Mazin et al., through a meta-analysis of multi-tissue transcriptomic data across different developmental stages, revealed that an intricate interplay of programs controlling gene expression levels and AS is fundamental to organ development [19]. Previously, based on second-generation RNA-seq data from cashmere goat hair follicles, Zhang et al. explored alternative splicing alterations, which showed distinct patterns among these three stages, finding that functional pathways of AS-regulated genes showed connections to hair follicle development [16]. In this study, we observed that among the three stages, exon skipping and alternative 5′ splice site events occurred most frequently during the Cn, while alternative 3′ splice site and intron retention events showed the opposite pattern, and mutually exclusive exon events occurred most frequently during the Tn. Additionally, compared to the Cn vs. Tn group, in certain genes related to lipid synthesis, metabolism, and storage (e.g., *CIDEA*, *SOAT1*, *ALDH3A2*, *GLYATL2*, *ECHDC1*, *DPM3*, *PLIN2*, *LOC108638299*, *LOC102186944*, *COL3A1*, etc.), a greater variety of transcript types were differentially expressed in the An vs. Cn group (Figure 2A). We hypothesize that from An to Cn, lipid synthesis-, metabolism-, and storage-related genes may enhance the expression of a broader range of transcript types, leading to an overall increase in their expression levels, thereby promoting fat deposition in skin tissue. Conversely, from Cn to Tn, the reduction in skin fat deposition may be achieved by decreasing the expression of some transcripts of these genes. These findings further support the potential influence of dynamic changes in the probability of alternative splicing events across different hair follicle developmental stages on the hair follicle cycle.

Notably, the analysis of differential AS events revealed that the alternative splicing patterns of the *ASCC1* and *SORL1* genes exhibited significant differences between both An vs. Cn and Cn vs. Tn, while the alternative splicing pattern of the *CEP19* gene showed significant differences only between An vs. Cn, involving three transcripts: ONT.23795.5 (*ASCC1*), ONT.14866.3 (*SORL1*), and ONT.911.3 (*CEP19*). Moreover, these two transcripts were significantly upregulated in the An vs. Cn group and significantly downregulated in the Cn vs. Tn group. This expression pattern is consistent with that of the vast majority of lipid metabolism-related genes. GO and KEGG enrichment analyses indicated that the *ASCC1* gene is associated with RNA binding, which plays a key role in post-transcriptional regulation of RNA expression, thereby suggesting the function of *ASCC1* gene [43]. Reports have also highlighted the critical role of *ASCC1* gene in DNA repair, and mutations in *ASCC1* gene may affect normal organ function or development, particularly in bones/muscles [44]. Given that both bone and hair follicles originate from pluripotent mesenchymal stem cells and share some molecular regulatory foundations [45,46], we further speculate that the alternative splicing pattern of *ASCC1* gene and the differential expression of its related transcripts may influence post-transcriptional RNA regulation, thereby correlating with changes in the hair follicle cycle. Considering the consistent expression patterns between ONT.23795.5 (*ASCC1*) and lipid metabolism-related DETs, we also reasonably hypothesize that *ASCC1* is involved in the regulatory expression of these DETs. Furthermore, the *SORL1* (Sortilin-related receptor 1) gene is associated with the endoplasmic reticulum membrane, nuclear envelope lumen, extracellular space, receptor-mediated endocytosis, and extracellular region. The endoplasmic reticulum is the site of lipid synthesis, indicating that *SORL1* gene not only plays important roles in maintaining cellular structure, spatial organization, communication, and recognition but may also influence lipid synthesis. Hair follicles are complex mini-organs composed of both ectoderm-derived epithelial cells and mesoderm-derived mesenchymal cells, and their growth and development require precise coordination and interaction among these cells. Lastly, some reports have also suggested that deletion or mutation of the *CEP19* gene is associated with obesity, a disease caused by dysregulation of lipid metabolism [47,48]. Therefore, given the consistency in expression patterns among ONT.14866.3 (*SORL1*), ONT.23795.5 (*ASCC1*), ONT.911.3 (*CEP19*), and DETs related to fat synthesis, metabolism, or storage, we also hypothesize that the alternative splicing patterns of *SORL1* and *CEP19* genes, along with the differential expression of their related transcripts, are associated with changes in the hair follicle cycle.

To further validate the findings of this study and strengthen the continuity and translational potential of the research, future experiments could focus on functional validation of the identified candidate DETs. For instance, in vitro assays such as gene knockout or overexpression in cultured dermal papilla cells or keratinocytes could be performed to examine the effects on lipid metabolism markers and hair follicle-related genes. Additionally, in vivo validation using transgenic or CRISPR-Cas9-edited animal models could help clarify the roles of key genes in regulating intradermal fat deposition and the hair follicle cycle. Furthermore, mechanistic studies could explore how alternative splicing variants of these genes influence downstream signaling pathways and their crosstalk with known follicle cycle regulators. Such efforts would not only confirm the regulatory functions of these candidates but also provide actionable targets for molecular breeding aimed at improving cashmere yield and quality.

## 5. Conclusions

This study provides a candidate DET profile (Tables S1–S3) related to hair follicle in Jiangnan cashmere goats based on ONT-seq data. The findings further support the close correlation between changes in intradermal fat deposition and the cyclical development of hair follicle, with DETs related to fat synthesis, metabolism, or storage playing important roles. The results also support the potential influence of dynamic changes in gene AS patterns on the hair follicle.

## Data Availability

The raw sequence data reported in this paper have been deposited in the Genome Sequence Archive (Genomics, Proteomics & Bioinformatics 2025) in National Genomics Data Center (Nucleic Acids Res 2025), China National Center for Bioinformation/Beijing Institute of Genomics, Chinese Academy of Sciences (GSA: CRA038669) that are publicly accessible at https://ngdc.cncb.ac.cn/gsa, accessed on 8 March 2026.

## Supplementary Materials

The following supporting information can be downloaded at: https://www.mdpi.com/article/10.3390/biology15060498/s1, Table S1: The DET analysis results of the An vs. Cn group; Table S2: The DET analysis results of the An vs. Tn group; Table S3: The DET analysis results of the Cn vs. Tn group; Table S4: The GO and KEGG enrichment analysis results of DETs in the An vs. Cn group; Table S5: The GO and KEGG enrichment analysis results of DETs in the An vs. Tn group; Table S6: The GO and KEGG enrichment analysis results of DETs in the Cn vs. Tn group; Table S7: The differential AS event analysis results of the An vs. Tn group; Table S8: The analysis results of the Cn vs. Tn group; Table S9: The differential AS event analysis results of the Cn vs. Tn group; Table S10: The interaction analysis results of DEGs, DEPs, and DEMs in the An vs. Cn group; Table S11: The interaction analysis results of DEGs, DEPs, and DEMs in the Cn vs. Tn group; genome.bed.
